# How do you see your role as a follower? A quantitative exploration of followers’ role orientation

**DOI:** 10.3389/fpsyg.2022.952925

**Published:** 2022-11-18

**Authors:** Elena Gesang

**Affiliations:** Chair of Business Administration, in Particular Work, Human Resource Management and Organization Studies, Heinrich Heine University Düsseldorf, Düsseldorf, Germany

**Keywords:** followership, followers’ role orientations, follower behavior, Implicit Followership Theories (IFT), latent profile analysis

## Abstract

How people see and define their role in different social settings has been of interest in psychological science for several decades. However, followers’ role orientations, followers’ beliefs about their role in the workplace, and how they execute their roles, have mostly been omitted in research so far. Nevertheless, followers’ role orientations are important as they can affect (work-related) behavior. Therefore, this study quantitatively investigates the structure, heterogeneity, and consistency of followers’ role orientations as well as the role orientations’ link to work-related traits and behaviors. For this purpose, content and statistical analysis of an Implicit-Followership-Theory Scale and latent profile analyses were conducted with data from two points in time via a sample of German employees (*t1*: *n* = 211, *t2*: *n* = 69). The results indicate that a passive/active work attitude (Enthusiasm) plays the most prominent part in differences in followers’ role orientation, followed by a positive/negative work ethic (Industry), and cooperativeness toward the leader (Good Citizen). Moreover, followers can be differentiated according to their role orientations into three distinct types: the Anti-Prototype, the Moderate Anti-Prototype, and the Moderate Prototype. Followers of the Moderate Prototype have the highest values in work-related traits and behaviors like conscientiousness and personal initiative. Followers’ profile affiliation is stable for three-quarters of the sample over 4–6 weeks. Overall, these findings point to role orientation being a (performance-related) follower characteristic, making role orientations relevant for application processes, especially for positions with frequent leader–follower interactions. Lastly, results show a current conceptual inaccuracy. They indicate the need to differentiate more rigorously between role orientations and Implicit Followership Theories (IFT), although currently both are often used synonymously. Therefore, recommendations for alterations to the used Implicit Followership Theory scale for capturing followers’ role orientations are given, including the elimination of items with situational character and changes in wording and factor structure.

## Introduction

How people see their differing, sometimes also conflicting, roles in both private and professional life has received considerable attention in psychological science (e.g., Graen, 1976; Hoover-Dempsey and Sandler, 1995; Parker, 2000; Green et al., 2007; Pinho and Gaunt, 2021). However, followers’ role orientations, being the beliefs followers have about their (hierarchically subordinate) role in relation to their leaders and how they execute their role, have been rarely investigated (e.g., Carsten et al., 2010, 2018).

A potential reason for this gap is that current research is rather leader-centric (e.g., Shamir, 2007; Agho, 2009; Uhl-Bien et al., 2014; Sturm et al., 2021; Tripathi, 2021), although it has been known for a long time that followers play a crucial part in the leader–follower relationship (e.g., Hollander and Webb, 1955; Zaleznik, 1965). For instance, followers can affect leaders’ behavior and state of mind and have an overall high share in the success of an organization (Herold, 1977; Podsakoff and MacKenzie, 1997; Carsten et al., 2018; Gesang and Süß, 2021).

Current research mainly considers followers’ role orientations as two (simplified) extremes, in which followers view their role as either (pro-)active or passive (e.g., Carsten and Uhl-Bien, 2013; Carsten et al., 2018). An active role orientation is characterized by believing that one is a co-creator of success, which includes feeling responsible for work achievements and feeling the need to contribute (e.g., Kelley, 1988; Shamir, 2007). This understanding can result in behavior such as expressing ideas of one’s own accord to the leader and taking on additional responsibilities (Carsten et al., 2010). Followers believing their role to be passive see their leaders as solely responsible and understand their role as just carrying out assigned tasks without thinking outside the box (e.g., Kelley, 1988; Shamir, 2007), which can result in followers just reacting and doing exactly what they are told without engaging any further (Carsten et al., 2010).

However, this dichotomous view is too general as followers’ role orientations are more complex (Carsten et al., 2010; Sy, 2010). First, (pro-)active and passive are rather at the extreme ends of a continuum, with followers also being somewhere in between (Carsten et al., 2010). Second, there seem to be more dimensions besides activity–passivity, as has already been addressed conceptually (e.g., Kelley, 1988, 2008), and empirically, in the thematically related field of Implicit Followership Theories (IFTs) (Sy, 2010) as well as in a first qualitative examination of followers’ role orientation (Carsten et al., 2010). Possible further dimensions include the degree of conformity or the degree of team orientation (Kelley, 2008; Sy, 2010). Researchers, thus, call for a(n) (quantitative) empirical exploration of followers’ role orientation, its heterogeneity, and its consequences for followers’ behavior (e.g., Carsten et al., 2010, 2018).

Consequently, the aim of this study is twofold: first, it is to explore (the content of) followers’ role orientations. Second, it is to identify how far followers differ with regard to their role orientations and their resulting behavior (regarding personal initiative, voice behavior, and helpfulness to colleagues).

For this purpose, data were collected at two points in time via a sample of German employees. Content and statistical analyses of an IFT scale (Sy, 2010) were conducted to adapt the scale to the purpose of measuring role orientations. Based on the adapted scale, latent profile analyses were generated, including a check of the stability of the followers’ latent profile affiliation.

This study offers several contributions to the literature on followership: first, it provides quantitative evidence for the heterogeneity of (the role orientation of) followers (Carsten et al., 2010). Second, it elucidates the link between followers’ role orientations and behaviors (e.g., Uhl-Bien et al., 2014). Third, it provides information about the link between personality traits and role orientation (Epitropaki et al., 2013). Fourth, it sheds light on the underlying structure of role orientation and its consistency (Sy, 2010). Fifth, it gives recommendations for the quantitative measurement of followers’ role orientation based on the IFT scale, to help tackle the issue that, to date, only very few validated scales exist that measure followership (e.g., Carsten et al., 2018).

## Conceptual background

One needs to consider Implicit Theories to understand the formation of role orientations (e.g., Shondrick and Lord, 2010; Kalish and Luria, 2021). Implicit Theories are socio-cognitive beliefs that people rely on to cope with life’s complexity, resulting in simplified classifications by ascribing stereotypical traits and skills to certain groups of people (Weiss and Adler, 1981; Lord et al., 1984).

In organizational settings, primarily two different groups can be differentiated, based on the group members’ hierarchical positions: leaders and followers (e.g., Levy et al., 2006; Epitropaki et al., 2013; Junker and van Dick, 2014). Consequently, IFTs and Implicit Leadership Theories exist as simplified assumptions about “the traits and abilities that characterize” followers or leaders (Epitropaki and Martin, 2004, p. 293). Based on their IFTs, followers shape their role orientation—sometimes also referred to as self-schema (Epitropaki et al., 2013) or follower beliefs (Carsten and Uhl-Bien, 2013)—meaning they shape beliefs about what their role is and how to enact it (e.g., Carsten et al., 2010, 2018).

Specific role orientations can only be formed and expressed if there is a clearly defined setting in which to enact the role (Carsten et al., 2010). This clearly defined setting applies to formal follower roles, as opposed to informal follower roles (e.g., Walter et al., 2012; Luria and Berson, 2013), with the former also being the most frequently considered roles in current research on followership and leadership (e.g., Bastardoz and Van Vugt, 2019; Blom and Lundgren, 2020). Formal follower roles are characterized by a hierarchically subordinate position in relation to the respective leaders. Leaders have exclusive resources that constitute their higher position and enable them to inform, incentivize, or put pressure on their followers (e.g., French and Raven, 1959; Yukl and Falbe, 1991; Farmer and Aguinis, 2005). These formal follower roles exist in different variants in organizations wherever people are hierarchically subordinate to an assigned leader, e.g., researchers in relation to their professors or consultants in relation to their managers (Uhl-Bien et al., 2014).

Followers’ role orientations evolve when the individuals are exposed to interactions in hierarchical relationships (e.g., Kuhn and Laird, 2011). These interactions can also include experiences early in life through hierarchical interactions with (early) caregivers, as has already been shown for experiences with one’s own parents with regard to certain Implicit Leadership Theories (Keller, 1999, 2003). It is, therefore, plausible to assume that also IFTs and, thus, a follower’s role orientation can be affected by parental imprint (Bastardoz and Van Vugt, 2019). Nevertheless, role orientations further develop when being exposed to the workplace and the relations therein (Hunt et al., 1990; Carsten et al., 2010; Kalish and Luria, 2021).

Apart from this rather general knowledge on the development of role orientations (also largely taken from the literature on Implicit Theories), expertise on the content and stability of followers’ role orientations is still scarce (Carsten et al., 2010, 2018), which is why a simplified (active versus passive) understanding of role orientation is currently predominant (Carsten and Uhl-Bien, 2012, 2013; Carsten et al., 2018). One rare exception is Carsten et al. (2010) qualitative study of followers’ role orientations. They discovered that followers’ role orientations can vary along a continuum from passive over active to proactive. Furthermore, they found that the respective role orientation can affect a follower’s behavior in various ways. It can have an impact on general work-related behavior, such as the effort the follower displays in everyday work. Moreover, due to followers’ primary reference point for interpreting their role, role orientations have implications for followers’ behaviors toward leaders, for instance, by affecting the offering of feedback, the voicing of ideas, as well as the showing of constructive resistance (Carsten et al., 2010, 2018; Carsten and Uhl-Bien, 2012, 2013). Finally, a follower’s role orientation can also affect colleagues, for example, via varying degrees of team orientation (Carsten et al., 2010).

If or to what extent a follower’s role orientation changes over time, is currently unclear. While it has been shown that Implicit Leadership Theories can be stable in time, considering 12 months (Epitropaki and Martin, 2004), the same has been demonstrated in a study examining IFTS, however, for a shorter period of only 3 months (Sy, 2010). The aforementioned Carsten et al. (2010) identified that followers whose role orientation is not in accordance with the workplace can experience stress and discomfort, rather than adopting their orientation to the environment, thereby indicating certain stability of role orientations. Nonetheless, there are also indications that a follower’s role orientation can change, for instance, when a naturally proactive follower gets a new, more authoritarian leader and tries to reduce the proactive execution of the role (Carsten et al., 2010).

Finally, it is important to specify the present and (apart from the specification to the follower-leader-relationship) rather broad understanding of role orientation, which sees role orientation as a set of attributes such as proactively thinking about problems (Carsten et al., 2018). However, it is neither “specific job behavio[u]rs” (Morrison, 1994, p. 1555), like whether a follower aims to come to work early (referred to as role definition; Morrison, 1994; Kamdar et al., 2006), nor is it about whether specific job aspects, like delivery times, are of the followers’ concern (understanding of role orientation of Parker et al., 1997; Howell and Boies, 2004; Parker, 2007). The present understanding is chosen because first, it provides a clear distinction between followers’ role orientation and followers’ behaviors, and second, it allows examining role orientations that may apply to followers in general by not being closely tied to the followers’ actual scope of activity.

## Materials and methods

For empirically investigating followers’ role orientations, the factor structure of role orientation was examined at two measurement occasions. This was done statistically via an analysis of item distributions, correlations, and a confirmatory factor analysis (CFA), as well as based on substantive arguments. Each time, a latent profile analysis (LPA) was conducted and profiles, being groups of followers, were identified that are as uniform as possible regarding their role orientations and as diverse as possible to the role orientations of the other profiles/the other groups of followers (Muthén and Muthén, 1998-2015). CFA and LPA were conducted with Mplus 7.4., while item distributions and correlations were analyzed with IBM SPSS Statistics 26.

Role orientation was based on Sy’s (2010) IFT scale. However, Sy’s scale was validated by external assessment. Leaders (and students, who can be considered as followers; Sy, 2010) were asked how characteristic 18 attributes (e.g., “productive”) are for a follower. Nonetheless, there is reason to assume that the same attributes apply to followers’ self-assessment of their role orientation. Research on self-schemas, which, as presented, can be seen as a synonym for role orientation, posits that people tend to use the same classifications for themselves as they ascribe to others (Catrambone et al., 1996). Since most leaders are also followers themselves (Collinson, 2006; Hackman and Wageman, 2007), it stands to reason that the same attributes apply to external as well as self-assessment. Additionally, the only existing empirical examination of followers’ role orientations (Carsten et al., 2010) points to some of the same attributes in the followers’ self-view as in Sy’s IFT scale (namely, the factors Conformity, Good Citizen, Industry, and the item “exited” of the Enthusiasm factor; Epitropaki et al., 2013).

To survey self-assessed role orientation, an introductory statement was generated which reads as follows: “To answer the following questions, please think of your role as an employee of your immediate (disciplinary) supervisor. To what extent do you agree with the statements?” The term “employee” was chosen because “follower” is uncommon in the German everyday language. However, the understanding of what a follower is was ensured by referring to the employee’s role in relation to the immediate disciplinary supervisor (German everyday language for “leader”). Moreover, because in German there is a feminine and masculine version of “supervisor,” participants were asked beforehand what gender their immediate disciplinary supervisor was. The corresponding word form was then used in the questionnaire.

The introductory statement was followed by “In my role as an employee, I see myself as …,” followed by a total of 17 randomly displayed attributes, e.g., “hardworking.” To ensure that the participants remained in their follower role, “In my role as an employee I see myself as…” was repeated each time after six attributes. The attributes of the IFT scale were translated into German and then back-translated by a bilingual German/English native speaker. For one attribute (*soft spoken*), there is no appropriate German translation. Closest would be “eine Person der leisen Töne” (literally “a person of soft tones”). Although this expression exists, it is highly uncommon in everyday language, which was confirmed during a discussion with 15 researchers (who conduct research in different fields of organizational behavior). Moreover, the bilingual native speaker emphasized that there is no German equivalent. Therefore, this attribute was excluded.

Consequently, role orientation was measured based on the described, adapted IFT scale. Participants responded on a 10-point Likert scale ranging from “fully disagree” to “fully agree.” If not reported otherwise, all other scales were measured on a 5-point Likert scale ranging from “fully disagree” to “fully agree.”

To better understand potential differences between the individuals in the different profiles, core self-evaluation traits (comprising self-esteem, self-efficacy, locus of control, and emotional stability; α = 0.87; Stumpp et al., 2010), as well as extraversion (α = 0.86), agreeableness (α = 0.75), and conscientiousness (α = 0.67; near, however, below the cut-off score of 0.7; due to the items being validated, and due to the importance of conscientiousness for work-related behavior, items stay included in the following analysis) of the Big Five personality traits (Rammstedt and John, 2005; measured on a 5-point Likert scale ranging from “very inapplicable” to “very applicable”) were collected, as they are relevant in explaining followers’ behavior and performance in the workplace (e.g., Judge and Bono, 2001; Thomas et al., 2010; Chiaburu et al., 2011).

Regarding behavioral outcomes, personal initiative (α = 0.80; Frese et al., 1997), voice behavior (α = 0.90; Liu et al., 2010), and helpfulness toward colleagues (a component of Organizational Citizenship Behavior; α = 0.76; Staufenbiel and Hartz, 2000; measured on a 7-point Likert scale ranging from “does not apply at all” to “applies completely”) were measured. These were selected to cover a wide range of behavior at work: personal initiative being of general importance for success at work (Hakanen et al., 2008), voice behavior being a vital indicator of followers’ behavior toward their leader (Walumbwa and Schaubroeck, 2009), and helpfulness toward colleagues covering behavior toward other followers. Additionally, age, gender, and education, whether the follower is also a leader, and how long the participant has been employed were collected.

Moreover, a two-item short scale measuring social desirability was included [ Satow, 2012; “I would never speak ill of a colleague or my employer”; “I have gossiped about others or thought badly of them before” (reverse)]. One item was mixed with the items of the aforementioned core self-evaluation traits scale. The other item was mixed with items of the Big Five scale, to make it harder for participants to recognize and outsmart the social desirability scale. To make the mixing of items possible, the originally 4-point scale, with a total sum of 7 and 8 to be considered a strong indicator for a participant to respond in a socially desirable way, got converted into a 5-point scale. Participants’ answers were then transformed back into the originally 4-point scale (e.g., with “1” equating to 0.8) and participants with a total sum higher than 6.65 were excluded (for a total view of all scales and items used, see Supplementary Table 3).

An online survey was conducted in 2019. Respondents were generated through professional contacts and by posting the questionnaire’s link on professional social networks. Although it is debated whether and how far incentives increase the likelihood of participation (e.g., Singer and Ye, 2013), participants were given the option to choose between either taking part in the raffle of two vouchers or having 50 cents donated to a charitable cause for their completed questionnaire (two options to reduce bias due to personal preference).

The sample size after controlling for implausibility and social desirability is *n* = 211 (*t*_1_). The sample consists of 63.51% females, the average age is 28.09 [Standard Deviation (SD) = 6.82] and 5.69% are also leaders themselves. Respondents were invited to participate in a follow-up questionnaire (time lag: 1 month). A total of 115 participants agreed to be contacted for this second survey. Anonymity was ensured via the generation of an anonymous code for each participant. One reminder was sent 2 weeks after the follow-up questionnaire. After controlling for implausibility, social desirability, and questionnaires filled in twice (only the first got considered), the sample size is *n* = 69 (*t*_2_). Sample *t*_2_ consists of 55.07% females, the average age is 31.26 (SD = 10.10) and 7.25% are also (disciplinary) leaders themselves. This data was used to re-examine the factor structure of role orientation (see: Re-examination of the factor structure of role orientation). After also controlling for unassignable anonymous codes, the sample size decreased to *n* = 51. This subsample consists of 52.94% females, has an average age of 31.76 (SD = 10.02), and 7.84% are also (disciplinary) leaders themselves. This data was used to check whether participants’ latent profile affiliation was stable in time (see: Check of the stability of the latent profile affiliation).

## Results

### Examination of the factor structure of role orientation

In the following, the factor structure of role orientation is examined to verify whether the factors identified by Sy (2010) can also be replicated in followers’ self-assessments of their role orientation.

**FIGURE 1:**
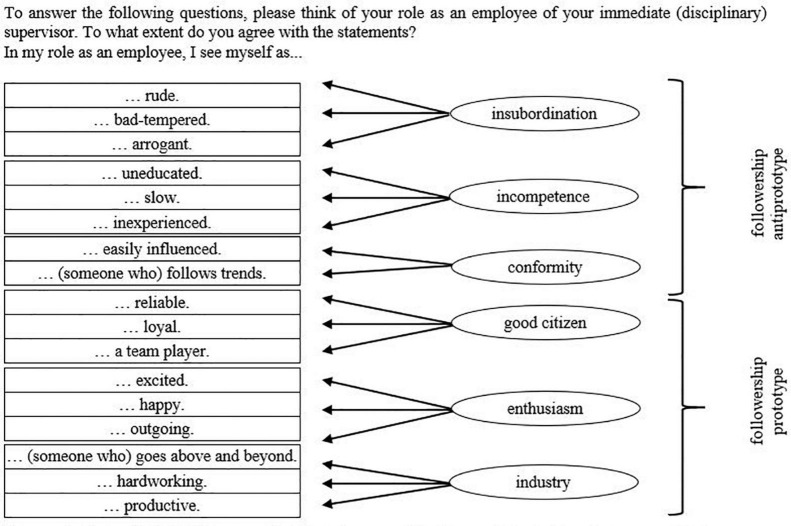
Sy (2010) Followership Prototype and Followership Anti-Prototype and their query in the study. “Soft spoken,” the third item of Conformity is not depicted, as it was not collected.

Figure 1 shows the second-order structure of Sy’s (2010) scale with three Followership Anti-Prototype factors (Insubordination: *rude, bad-tempered, arrogant*; Incompetence: *uneducated, slow, inexperienced*; Conformity: *easily influenced, follows trends*), as well as three Followership Prototype factors (Good Citizen: *reliable, loyal, team player*; Enthusiasm: *excited, happy, outgoing*; Industry: *goes above and beyond, hardworking, productive*), and the statement that introduced the items in the questionnaire.

The distribution of two of the collected items was salient. *Rude* as well as *uneducated* showed nearly no variance, with 77.73% (*rude*) and 72.99% (*uneducated*) of the followers answering with a 1 or 2 on the 10-point scale, indicating that followers might not see their role as being rude or uneducated.

Regarding *rude*(ness), research on workplace incivility indicates that incivility is likely to be triggered by, among other things, job dissatisfaction, felt injustice, and work exhaustion (Blau and Andersson, 2005). These aspects underscore the situational character of rudeness, which can be a reaction to negatively perceived circumstances (see also Schilpzand et al., 2016). Moreover (although the consequences of being rude in the workplace have not been extensively investigated; Schilpzand et al., 2016), being rude can lead to being excluded by colleagues (Scott et al., 2013), therefore leading to unpleasant consequences in the workplace. The situational character, as well as the possible negative consequences, make it unlikely that followers per se define their role as being rude.

Concerning *uneducated* it is most likely that followers do not see themselves as uneducated, as usually, followers are only in their respective position if they are qualified for the job. Although followers may differ in their education, a follower’s role orientation does not aim at a comparison either with colleagues or with the leader. While there may be circumstances, such as a(n) (extreme) lack of qualified staff (e.g., Beechler and Woodward, 2009) or obtaining a job based on contacts/personal favors, where a follower may not be adequately qualified, these circumstances usually do not occur frequently. Additionally, and most importantly, it is very unlikely that a follower under these circumstances sees the follower role as being uneducated, sticks to that, and refuses to improve and learn. If a follower had such a role orientation, it would instead be very likely that the follower would have (severe) difficulties at work due to low performance and would have to adjust the understanding of the follower role in the long run.

Bivariate correlations (for a total view of all correlations in *t*_1_, see Supplementary Table 1) showed that the two remaining items of the Insubordination factor *arrogant* and *bad-tempered* had only a very weak (linear) relationship with each other (considering here and in the following ≤0.30 a very weak relationship; correlation: 0.16). This might be due to while arrogance is a trait (e.g., Meagher et al., 2015), being *bad-tempered* is a situational mood (which additionally makes it less likely to be a component of a role orientation). As *bad-tempered* and *rude* (see above) share their situational character and also their negative connotation, here is a possible explanation why *bad-tempered* showed variance, while *rude* did not. Being bad-tempered can but does not necessarily translate to interaction with leaders and colleagues, while being rude is characterized by being rude to another person (e.g., Vahle-Hinz et al., 2019). Therefore, it is likely that followers stated varying degrees of seeing themselves as bad-tempered, as it does not necessarily affect another person in a bad way.

A slightly higher but still weak statistical relationship (correlation: 0.22) is found between the two conformity items *easily influenced* and *follows trends*. While in a follower’s role orientation, being directly aligned to a follower’s role in relation to the leader, the former one is directly assigned to the influence of the leader, *follows trends* is a much looser reference, due to the rather abstract “trends.” While these two items may work when examining IFTs (as although followers are still defined by their lower hierarchical position, the leader is not omnipresent in the sole term “follower”), for followers’ role orientation the direct versus abstract reference seems to make a relevant difference.

Additionally, the two remaining items *slow* and *inexperienced* of the Incompetence factor also showed a weak relationship (correlation: 0.28). Instead, *slow* seemed to be related to *productive* (correlation: -0.50), which is plausible as productivity is usually understood as the amount of work one can do, given a particular period of time (Cambridge University Press, 2014a). That *slow* did not fit well with *inexperience* is reasonable as a relation is most probably in the way that an inexperienced follower tends to be slower than an experienced one. However, this understanding (again) implies a situational component and does not apply when estimating the general understanding of one’s follower role.

Lastly, bivariate correlations revealed that the item *reliable* only added little to the understanding of the factor Good Citizen (both correlations <0.3). From a content perspective, this is plausible since the items “team player” and “loyal” focus exclusively on relationship aspects between people, whereas “reliable” can refer to people (to rely on someone) or to objects and also tasks, like when a follower performs tasks in a reliable way (Cambridge University Press, 2014b). In German, there are two different terms for this distinction. However, both their English translations are “reliable”. It is “zuverlässig” being for people as well as objects/tasks, and “verlässlich” (close to “trustworthy”) being for people only (Cornelsen Verlag GmbH, 2022a,b). Because the former is more commonly used in the work context, this term was used in the present study.

To summarize, there is both statistical and content evidence that (1) followers see themselves as neither *rude* nor *uneducated*. (2) *Arrogant* and *bad-tempered* do most certainly not form one factor. The same applies to *easily influenced* and *follows trends* as well as to *slow* and *inexperienced*. (3) Reliable probably does not fit the Good Citizen factor.

The now remaining factors Good Citizen, Enthusiasm, and Industry have also (except for *happy*) been identified in Carsten et al.’s (2010) qualitative study of followers’ role orientations (see also: Epitropaki et al., 2013). They found that followers varied in how far they saw their role as being a team player and loyal to their leader (Good Citizen factor) (Carsten et al., 2010). Followers also differed in how far they regarded their role as showing personal initiative, taking over responsibilities, and voicing their own ideas (Industry factor, and excited and outgoing of the Enthusiasm factor; Epitropaki et al., 2013) (Carsten et al., 2010). Although *happy* was not identified in Carsten et al.’s (2010) study and is also rather situational, making it less suitable for role orientations, it remains included in the following analysis due to the statistical reason of avoiding two two-item factors.

**FIGURE 2:**
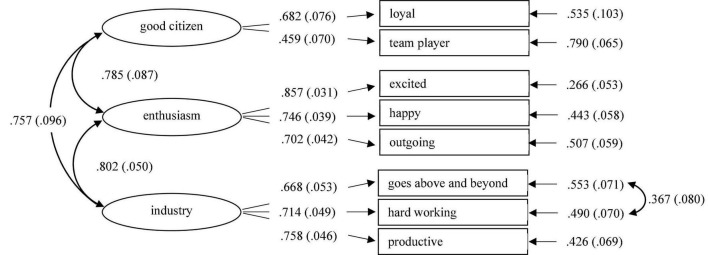
Confirmatory factor model (Followership prototypes). Standardized parameters, standard errors are given in brackets.

A CFA was performed using the three remaining Followership Prototype factors, the results of which are depicted in Figure 2.

The items *go above and beyond* and *hardworking* were allowed to load on each other (as they were the only items that indicated an overfulfillment of follower requirements), while all other items were exclusively allowed to load on their own factors. Although the Chi-Square Test of Model Fit was significant [χ^2^ (16) = 29.945, *p* = 0.018], the other indicators proved the model to have a very good fit with Root Mean Square Error of Approximation (RMSEA) = 0.064, Comparative Fit Index (CFI) = 0.978, Tucker–Lewis Index (TLI) = 0.962, and Standardized Root Mean square Residual (SRMR) = 0.034.

### Latent profile analysis of followers’ role orientation

Based on the proposed model, an LPA was conducted (Muthén and Muthén, 1998–2015; Nylund-Gibson and Choi, 2018). Latent profiles (one to ten) were generated based on the means, which is a common and accepted procedure (e.g., Mäkikangas et al., 2018). For means and variances, Maximum Likelihood estimation with Robust standard errors (MLR) estimator was used, as were 10,000 random starts with 1,000 final stage optimizations (or final “picks”) and 100 initial stage iterations for the generation of the latent profile solutions. Table 1 shows the statistical results of the analysis.

**TABLE 1:** Statistical results from the latent profile analysis (*t*_1_).

Model	LL	#fp	AIC	BIC	aBIC	AWE	cmP	Entropy
(1) Profile	−1106.485	6	2224.971	2245.082	2226.07	2239.42	0.000	NA
(2) Profiles	−1043.078	10	2106.156	2139.674	2107.988	2130.23	0.000	0.776
(3) Profiles	−1017.454	14	2062.908	2109.834	2065.473	2096.63	0.992	0.783
(4) Profiles	−1011.576	18	2059.153	2119.486	2062.451	2102.50	0.008	0.83
(5) Profiles	−1006.021	22	2056.041	2129.782	2060.073	2109.03	0.000	0.735
(6) Profiles	−1001.529	26	2055.058	2142.206	2059.822	2117.68	0.000	0.742
(7) Profiles	−996.433	30	2052.866	2153.422	2058.363	2125.12	0.000	0.825
(8) Profiles	−990.522	34	2049.045	2163.008	2055.275	2130.93	0.000	0.808
(9) Profiles	−983.839	38	2043.678	2171.049	2050.641	2135.20	0.000	0.834
(10) Profiles	−978.752	42	2041.504	2182.282	2049.2	2142.66	0.000	0.856

LL, model log-likelihood; #fp, number of free parameters; AIC, Akaike information criterion; BIC, Bayesian information criterion; aBIC, (samplesize) adjusted BIC; AWE, approximate weight of evidence criterion; cmP, correct model Probability.

The analysis revealed three latent profiles to be the best solution. Bayesian information criterion (BIC) (2109.834) and approximate weight of evidence criterion (AWE) (2096.63) showed minima, indicating the best solution, and the correct model Probability (cmP) (0.992) indicated that this model was the most likely to be correct (in comparison to the other nine models). In addition, the three and four-latent-profile models were checked for statistically significant differences in the three considered factors. While with the three-latent-profile model, all role orientation factors were statistically significant, with the four-latent-profile model, there were non-significant differences. The three-latent-profile solution was robust to changes in the number of random starts and the number of final “picks” for 100,000 and 2,000 as well as 200,000 and 10,000.

The grand mean and standard deviation, as well as the means of Good Citizen, Enthusiasm, and Industry for the three latent profiles, and the profiles sample sizes are depicted in Table 2.

**TABLE 2:** Mean levels of role orientation factors in the three-latent-profile solution (*t*_1_).

	Grand mean	SD	Profile 1 (*n* = 9)	Profile 2 (*n* = 79)	Profile 3 (*n* = 123)
Good citizen	8.047	1.260	5.644	7.480	8.607
Enthusiasm	6.956	1.612	3.235	5.966	7.894
Industry	7.730	1.321	4.707	7.034	8.422

*n* = 211; SD, standard deviation.

**FIGURE 3:**
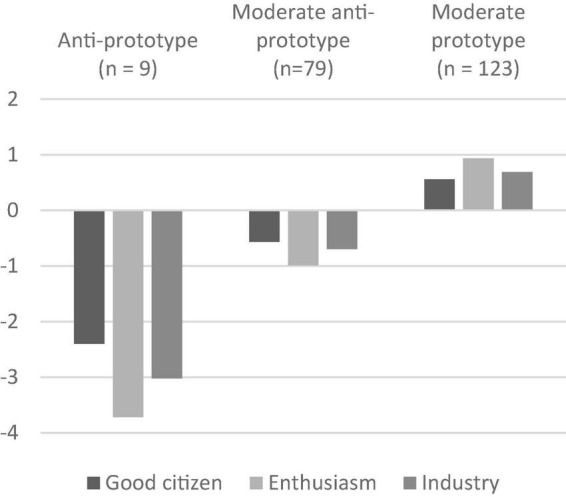
Profiles of the three-latent-profile solution (*t*_1_). Zero line is the grand mean.

Figure 3 shows the latent profiles. All profiles have the same structure of underlying factors, in the way that Enthusiasm is the (positively or negatively) dominating factor, followed by Industry, and then Good Citizen [Additional note: To ensure that this result was not due to a relatively high correlation of the Enthusiasm item *exited* with the three Industry items (Pearson Correlations of 0.49, 0.50, and 0.54), the profiles were again generated with exclusion of this item. The structure of the profiles stayed the same].

The first profile has the strongest below-average manifestations of all three followership prototype factors. Therefore, it is labeled Anti-Prototype. The second profile is characterized by more positive, but still below average, manifestations of the factors and is thus named Moderate Anti-Prototype. The third profile also has moderate, however, above-average manifestations of the factors and is therefore labeled Moderate Prototype.

The Anti-Prototype profile consisted of four male and five female followers, average age was 26.33 (SD = 5.148), and the followers had been employed for an average of 3.11 years (SD = 2.37). These characteristics made the Anti-Prototype profile the youngest (however, age was not statistically significant) and the least professionally experienced (significant: *p*-values: 0.04 and 0.03) profile.

The Moderate Anti-Prototype profile consisted of 59.49% female followers. The average age was 28.92 (SD = 8.52), and the followers had been employed for an average of 5.59 years (SD = 7.69). This profile was the oldest and most professionally experienced (although both variables were non-significant).

The Moderate Prototype profile consisted of one non-binary follower, 82 female, and 40 male followers. The average age was 27.67 (SD = 5.56), and the followers had been employed for an average of 5.19 years (SD = 5.56).

Subsequently, the profiles were tested for significant mean differences in traits as well as behaviors. The results are shown in Table 3.

**TABLE 3:** Differences in means for traits and behaviors of the three latent profiles (*t*_1_).

		Anti-prototype (1)	Moderate anti-prototype (2)	Moderate prototype (3)	Significant order
Traits	Agreeableness	2.555	2.878	3.131	n.s.
	Conscientiousness	3.444	3.662	4.091	1 = 2 < 3
	Extraversion	2.471	3.010	3.832	1 = 2 < 3
	Core self-evaluation traits	3.166	3.248	3.865	1 = 2 < 3
Behaviors	Personal initiative	3.079	3.400	4.082	1 < 2 < 3
	Voice behavior	2.356	3.142	3.545	1 < 2 < 3
	Helpfulness toward colleagues	2.663	5.043	5.716	1 < 2 < 3

*n* = 211; Differences based on auxiliary 3-step “BCH” approach with χ^2^-tests of equality between latent profiles; n.s., no significant differences across all latent profiles.

Agreeableness was not significantly different across all profiles. However, followers of the Moderate Prototype profile had the significantly highest levels of conscientiousness, extraversion, and core self-evaluation traits compared to the other profiles.

Concerning behaviors, all profiles were statistically different from each other. Followers of the Anti-Prototype profile had the lowest scores on personal initiative, voice behavior, as well as helpfulness toward colleagues, followed by the followers of the Moderate Anti-Prototype profile, while followers of the Moderate Prototype profile had the significantly highest levels on these behaviors.

### Check of the stability of the latent profile affiliation

To check the stability of the latent profile affiliation, profiles were generated again with the subsample of *t*_2_ (*n* = 51). The statistical analysis was not as unambiguously clear as in *t*_1_. BIC and AWE were close for the one to the three-profile solution. However, BIC showed a minimum of three profiles, while AWE showed a minimum of one profile, and cmP indicated four profiles to be the best solution, as shown in Table 4.

**TABLE 4:** Statistical results from the latent profile analysis (*t*_2_).

Model	LL	#fp	AIC	BIC	aBIC	AWE	cmP	Entropy
(1) Profile	−264.689	6	541.378	552.969	534.131	555.83	0.126	NA
(2) Profiles	−257.882	10	535.765	555.083	523.687	559.85	0.044	0.725
(3) Profiles	−247.120	14	522.24	549.285	505.331	555.96	0.795	0.849
(4) Profiles	−242.392	18	520.785	555.558	499.045	564.14	0.850	0.85
(5) Profiles	−239.154	22	522.308	564.808	495.737	575.29	0.000	0.862
(6) Profiles	−236.724	26	525.449	575.676	494.047	588.07	0.000	0.907
(7) Profiles	−229.000	30	518.043	575.998	481.81	590.30	0.000	0.968
(8) Profiles	−224.145	34	516.29	581.972	475.226	598.18	0.000	0.979
(9) Profiles	−218.570	38	513.14	586.549	467.245	604.66	0.000	0.969
(10) Profiles	−213.593	42	511.186	592.322	460.46	612.34	0.000	0.856

LL, model log-likelihood; #fp, number of free parameters; AIC, Akaike information criterion; BIC, Bayesian information criterion; aBIC, (samplesize) adjusted BIC; AWE, approximate weight of evidence criterion; cmP, correct model Probability.

However, rather small sample sizes can lead to statistical fit indices being less reliable (Nylund-Gibson and Choi, 2018). Due to the importance of drawing on well-separated classes, especially when having a small sample size (Nylund-Gibson and Choi, 2018), the two-, three-, and four-profile solutions were checked for significant differences in the role orientation factors. The two-profile solution was the only one with all profiles being significantly different from each other and was therefore chosen. This solution was robust to changes in the number of random starts and the number of final “picks” (100,000 with 2,000, and 200,000 with 10,000).

Table 5 shows the grand mean, standard deviation, means of the role orientation factors, and the profiles’ sample sizes.

**TABLE 5:** Mean levels of role orientation factors in the two-latent-profile solution (*t*_2_).

	Grand mean	SD	Profile 1 (*n* = 26)	Profile 2 (*n* = 25)
Good citizen	7.765	1.856	7.448	8.105
Enthusiasm	6.627	2.369	5.447	7.896
Industry	7.830	1.470	7.321	8.377

*n* = 51; SD, standard deviation.

**FIGURE 4:**
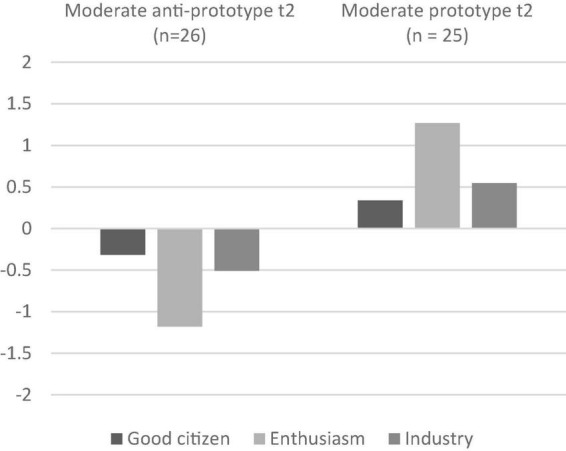
Profiles of the two-latent-profile solution (*t*_2_). Zero line is the grand mean.

Figure 4 shows the latent profiles. All profiles have (again) the same structure with Enthusiasm still being the dominating factor, followed by Industry, and last Good Citizen. Although the elevations vary a bit (which is most certainly due to the smaller sample and, therefore, a different mean), the manifestations can now as before be described as Moderate due to the highest deviations from zero being around 1, resulting in a Moderate Anti-Prototype profile and a Moderate Prototype profile.

Regarding the latent profile affiliation, 24 of the 51 followers who participated in *t*_2_ had been in the Moderate Anti-Prototype profile in *t*_1_, while 27 had been in the Moderate Prototype profile in *t*_1_. It is not surprising that no follower who had been in the Anti-Prototype profile in *t*_1_ participated in the second survey. This is because the profile was small (although statistically, two followers would have participated if participation had been equally distributed across all profiles) and these followers had the significantly lowest levels of personal initiative, which may be an additional reason. About 76.47% of the followers remained in their previous profile.

Twelve followers changed their profile (five from Moderate Anti-Prototype to Moderate Prototype and seven vice versa). A closer analysis of these 12 followers was conducted, taking the class affiliation probabilities into account. These indicate the probability with which a follower belongs to a particular profile. In *t*_1_ for instance, the average probability with which followers were assigned to their profile was 0.90 (Median: 0.97; SD: 0.13) The analysis revealed that four followers already had been rather closely assigned to their profile in *t*_1_ with their probabilities ranging from 0.57 to 0.68, while three followers had been very closely assigned to their profile in *t*_2_ (probabilities between 0.57 and 0.62). These seven followers (13.37%) could therefore not be assigned to profiles as clearly as others, which also explains the switching between profiles. However, no reason could be found for the five remaining followers (9.80%) who also switched profiles.

About 53.8% of the followers in the Moderate Anti-Prototype profile (*t*_2_) were male, the average age was 31.81 (SD = 11.32), and the followers had been employed for an average of 7.54 years (SD = 8.51).

Regarding the Moderate Prototype profile (*t*_2_), 60% of the followers were female, the average age was 31.72 (SD = 8.71) and the average time of employment was 7.28 years (SD = 9.21).

In the next step, the profiles were tested for significant mean differences in traits and behaviors (α = 5%). The results are shown in Table 6. Except for voice behavior that showed no significant difference, all findings from *t*_1_ were replicated: agreeableness was (again) not significantly different. Followers of the Moderate Prototype profile (*t*_2_) had the significantly highest levels of conscientiousness, extraversion, core self-evaluation traits, personal initiative, and helpfulness toward colleagues.

**TABLE 6:** Differences in means for traits and behaviors of the two latent profiles (*t*_2_).

		Moderate anti-prototype (1)	Moderate prototype (2)	Significant order
Traits	Agreeableness	2.889	3.024	n.s
	Conscientiousness	3.653	4.129	1 < 2
	Extraversion	2.690	3.964	1 < 2
	Core self-evaluation traits	3.409	3.835	1 < 2
Behaviors	Personal initiative	3.444	3.987	1 < 2
	Voice behavior	3.158	3.291	n.s.
	Helpfulness toward colleagues	5.272	5.838	1 < 2

*n* = 51; Differences based on auxiliary 3-step “BCH” approach with χ^2^-tests of equality between latent profiles; n.s., no significant differences across all latent profiles.

### Re-examination of the factor structure of role orientation

In the following, it is re-examined whether the findings on the factor structure of role orientation in *t*_1_ also occur in *t*_2_.

Regarding the finding of *t*_1_ that followers do not see themselves as *rude* and *uneducated* (1), sample *t*_2_ supports that. About 78.26% answered with a 1 or 2 on the 10-point-scale for *rude* (*t*_1_: 77.73%) and 73.91% answered with a 1 or 2 for *uneducated* (*t*_1_: 72.99%).

The same is valid for (2), that *arrogant* and *bad-tempered* do not form one factor (correlation: 0.23, *t*_1_: 0.16). The items *easily influenced* and *follows trends* showed a slightly different, however, still just about acceptable correlation in *t*_2_ of 0.33 (*t*_1_: 0.22; considering >0.30 acceptable as in *t*_1_). There was no indication that the higher correlations could be due to skewed items and/or the smaller sample size, as the distribution properties of the items had been checked in advance. *Slow* and *inexperienced* correlated stronger and acceptable this time at 0.38 (*t*_1_: 0.28), albeit *slow* still correlated more strongly with *productive* (-48; *t*_1_: −0.50).

Regarding that (3) *reliable* does not fit the Good Citizen factor, *t*_2_ supported this with correlations with the other items of <0.30 (as in *t*_1_). For a total view of all correlations in *t*_2_, see Supplementary Table 2.

As in *t*_1_, a CFA with the three remaining factors was performed. The model showed a worse, albeit acceptable, fit compared to the model in *t*_1_. The Chi-Square Test of Model Fit was again significant [χ^2^ (16) = 29.650, *p* = 0.020] and the other indicators proved the model’s acceptable fit with CFI = 0.929, TLI = 0.876, SRMR = 0.068, and RMSEA = 0.110.

## Discussion

### Implications of the findings regarding followers’ role orientations

This study reveals Enthusiasm, followed by Industry, and last Good Citizen to have the largest manifestations in differences in followers’ role orientations. Considering that high levels of Enthusiasm and high levels of Industry can be seen as representing an active role orientation, while low levels of both can be viewed as a passive role orientation (Epitropaki et al., 2013), the findings indicate that followers’ role orientations indeed primarily differ in (pro-)activity and passivity, thereby supporting the (qualitative) findings of Carsten et al. (2010). Nevertheless, of importance are also relational aspects (Good Citizen) that do not fit into the activity–passivity continuum. That relational aspects matter again supports Carsten et al. (2010), who found that followers differ in how far their role orientations include being loyal and a team player. Against the background of the relatively small sample size (*n* = 31) of Carsten et al. (2010) study and their call for replications with various methodologies, this study strengthens the legitimacy of their findings based on a much larger population.

However, apart from this (quantitative) replication of Carsten et al. (2010) findings, this study also further deepens the understanding of followers’ role orientations. The findings show that Enthusiasm, Industry, and Good Citizen can clearly be differentiated and have a hierarchy in their explanatory power for differences in followers’ role orientations. Enthusiasm can be considered as a “work attitude” that can be located along an activity–passivity continuum, meaning that followers have a rather enthusiastic (active) or rather non-enthusiastic (passive) attitude toward their work (environment), including the leader (De Hoogh et al., 2005). Industry shows the dedication to work hard and thereby comprises, in contrast to Enthusiasm, an explicit performance component. Industry thus describes a positive/negative “work ethic—a commitment to the value and importance of hard work” (Miller et al., 2002, p. 452). Lastly, Good Citizen can be seen as a form of “cooperativeness toward the leader.” Future research may benefit from concretizing and expanding the understanding of followers’ work attitude and performance-related work ethic, to further concretize followers’ activity–passivity, as well as from specifying followers’ cooperativeness toward the leader with additions to the current attributes (team player, loyal).

While role orientations were shown to be stable for three-quarters of the participating followers over 4–6 weeks, there are plausible reasons to assume that especially followers’ Enthusiasm and Good Citizen(ship) may be affected by leaders or the followers’ work environment. Followers’ work attitude (Enthusiasm) is positively related to charismatic leadership (De Hoogh et al., 2005). This is especially plausible as leader behavior has been shown to (causally) affect follower attitudes, for example, job satisfaction (Skogstad et al., 2014). Additionally, followers’ “cooperativeness toward the leader” (Good Citizen) may be particularly affected by the contextual, sometimes temporary, quality of the leader–follower relationship. This is because the extent to which a follower is willing to cooperate can depend on the perceived trustworthiness and honesty of the respective leader (rule of reciprocity; Van de Calseyde et al., 2021). Consequently, especially Enthusiasm and Good Citizen may be important when future research examines (potential) (in-)consistencies in followers’ role orientations over longer periods. When examining these, research may consider additional variables like “change in leader-(ship),” “(change in) perceived trustworthiness of the leader,” “critical incidents with the leader,” or such, to shed light on causes for (potential) changes in role orientations.

Apart from this, there are still additional reasons to look for within the followers themselves when further examining the stability of followers’ role orientations. One is an examination of followers’ implicit person theories (IPTs), beliefs about the extent to which personal characteristics are (un-)changeable (Seitz and Owens, 2021). IPTs can be a means to explain why some followers may have a very stable role orientation, even under changing circumstances, while other followers may not.

Jointly considering followers’ role orientations, traits, and behaviors, the study identified three clearly distinct profiles (Anti-Prototype, Moderate Anti-Prototype, and Moderate Prototype). Members of the profiles (i.e., followers) differ in their role orientations and behavior (with voice behavior being only significantly different in *t*_1_) and partially in traits. The followers of the Moderate Prototype have the highest values in the collected items of role orientation, indicating that they see their role as having an active “work ethic,” an active “work attitude,” and a high “cooperativeness toward the leader.” This understanding of their role is in accordance with them having the highest (self-reported) values in proactive behavior, as personal initiative, voice behavior, and helpfulness toward colleagues all can be considered proactive (Frese et al., 1997; Parker et al., 2019) or non-incentivized (and therefore proactive) behavior (Organ, 1997). Furthermore, the members had at both points in time the significantly highest values in collected traits of which especially conscientiousness can be seen as valuable in the workplace (e.g., Barrick and Mount, 1991).

However, although in this study named Moderate-*Proto*type, which is in line with a tendency in research and practice to view followers’ proactivity positively (e.g., Thomas et al., 2010; Morrison, 2014; Chamberlin et al., 2017), not all leaders may favor a rather active understanding of the follower role. This phenomenon has been labeled “the initiative paradox,” which describes a follower’s proactivity only to be positive as long as the proactivity is in line with the leader’s expectations (Campbell, 2000). A general fit between leaders’ (and followers’) expectations and followers’ (and leaders’) behaviors is moreover likely to positively affect the leader–follower relationship (e.g., van Gils et al., 2010). This is why it may be relevant to capture how followers see their role when they are applying for a position with frequent leader–follower interaction (e.g., assistant to the management), to select an applicant with a degree of proactivity that is in line with the respective leader’s expectations. Potential means to capture followers’ role orientations in application processes may be situational judgment tests (McDaniel et al., 2007; Bledow and Frese, 2009). In these tests, applicants are presented with descriptions of work-related situations. They then need to select how they would most likely react from a given number of options, or they need to rank different options according to their likeliness (McDaniel et al., 2007; Bledow and Frese, 2009). For assessing a follower’s role orientation, the work-related situation should concern the applicant’s (i.e., potential follower’s) understanding of his/her role respectively his/her behavior toward the leader. For instance, the work-related situation could include a (potential) follower’s option to behave in an outgoing way toward the leader. The (potential) follower then could be given several options to choose or rank how s/he would behave. The options should cover varying degrees of outgoingness to depict a rather high level (i.e., active) and a rather low level (i.e., passive) of outgoingness.

### Recommendations for the quantitative measurement of followers’ role orientation

The study furthermore demonstrates that there is a difference between “Followers’ social constructions of the follower role” (IFTs) and “Followers’ social constructions of *their own* [follower] roles” (role orientations; Coyle and Foti, 2022, p. 125). Both points in time indicate that, for instance, some characteristics (e.g., *uneducated*) found to be part of (leaders’ and) followers’ IFTs (Sy, 2010) are not part of followers’ role orientations. This stresses the importance of differentiating both concepts (and, concerning the measurement, the importance of establishing two related but separate constructs). However, in current research, the terms IFT and role orientation are often used synonymously (Epitropaki et al., 2013; Junker and van Dick, 2014), or role orientations are referred to as IFTs (Coyle and Foti, 2022). A similar aspect concerns concepts/constructs closely related to role orientations, such as follower beliefs. Although follower beliefs are referred to as how followers “see their role” (Carsten and Uhl-Bien, 2012, p. 211; which is why they were considered synonymous with role orientations; see: Conceptual background), their measurement refers to the follower role in general, as an exemplary item shows: “Followers should be on the lookout for suggestions they can offer to superiors” (Carsten and Uhl-Bien, 2012, p. 214). The present study indicates that there is a need to concretize the measurement to the own specific follower role when wanting to gain insights into how follower see their own (specific) role. This is especially relevant as both, followership and leadership literature, in parts lack clear conceptual understandings (e.g., Andersen, 2019; Alvesson, 2020). Therefore, recommendations for the quantitative measurement of followers’ role orientation are provided in the following.

The present findings lead to the following conclusion when wanting to draw on Sy’s (2010) IFT scale for capturing followers’ role orientation: being *rude* or *uneducated* are, in all probability, not part of followers’ role orientations. While *reliable*, *arrogant*, and *bad-tempered* may have a part in followers’ role orientations, they do not belong to the factor Good Citizen respectively do not form the factor Incompetence. Regarding the term “reliable,” one solution may be to use trustworthy instead (or “verlässlich” instead of “zuverlässig” in German). As to *bad-tempered*, it may be helpful not to use this item due to its situational character, thereby contradicting the chances of surveying a general understanding of a follower’s role.

The findings are ambiguous regarding whether *easily influenced* and *follows trends* form one factor (Conformity factor). However, even in the best case (*t*_2_) the correlations are close to being not acceptable. Overall, the difference in content regarding the direct reference to a leader’s (potential) influence on a follower via *easily influenced*, and, in contrast, the rather abstract reference via *follows trends*, with no direct reference to the specific leader-follower relationship, is likely to cause difficulties. Nevertheless, the qualitative study by Carsten et al. (2010) indicates that following the “leader’s way” (Carsten et al., 2010, p. 556) is an important characteristic to capture particularly passive role orientations (see also: Epitropaki et al., 2013). Therefore, it may be especially fruitful not to disregard these items/this factor, but to try to create additional items (ideally as an addition to *easily influenced* due to its direct reference) to capture the degree of conformity of a follower’s role orientation.

Concerning *slow* and *inexperienced*, the correlations in *t*_2_ were acceptable. However, *slow* both times correlated stronger with *productive*. Additionally, there is (again) the difficulty with the situational character of *inexperience*, which is why it may be useful to exclude *inexperience* entirely. Moreover, it could be a solution to form a new factor, consisting of *productive* and *slow* (reversed), that could be named “efficiency.” *Productive* could then no longer be part of the factor Industry. However, the remaining items of the factor (*goes above and beyond*, *hardworking*) have indeed a higher connotation of diligence than *productive*, which is why a separation may be sensible. To avoid potential problems due to two-item measures, the generation of additional items may be helpful.

Finally, one note regarding the item *happy* (Enthusiasm factor). It is (also) very situational and, therefore, not well suited for capturing role orientations [which may be a reason why happiness was no relevant characteristic in Carsten et al.’s (2010) qualitative study]. *Happy* also showed relatively high correlations with items of the Industry factor. Nevertheless, it remained in this study to avoid having two two-item measures, although the exclusion of *happy* did not lead to different profiles anyway. Overall, it may be useful to replace *happy* with a less situational and less overlapping term.

In summary, the following recommendations can be given: first, excluding any items with situational character is sensible (*happy, inexperienced, and bad-tempered*). Second, items with a direct reference to the leader-follower relationship (e.g., *easily influenced*) should be prioritized over items with an indirect reference (e.g., *follows trends*). Items with a direct reference should also not be mixed with items with an indirect reference in the same factor. Third, creating a factor called “efficiency” comprising *productive* and *slow* is plausible. Fourth, *rude* and *uneducated* can most likely be dropped from any measurement of followers’ role orientations. Fifth, to broadly capture followers’ role orientations in the future, more items, especially some that capture passive role orientations, need to be generated.

### Limitations

The small sample size in *t*_2_ is a major limitation of this study. The sample used for re-examining the factor structure of role orientation can be considered adequate (Cattell, 1978; MacCallum et al., 1999), although there is no consensus on sample sizes for confirmatory factor analyses. Nunnally (1978), for instance, recommends a sample size of *n* = 80 (for eight items) instead of *n* = 69 here. However, the subsample of *t*_2_ is very small. This is why especially the stability of the latent profile affiliations, and the (potentially causal) links between followers’ role orientation and behavior need further investigation.

In addition, causality cannot be derived from the present study. Although the (with one exception: voice behavior) consistency in both points in time in role orientations, traits, and behaviors of the Moderate Prototype and the Moderate Anti-Prototype is an indicator of a causal relationship, a Latent Transition Analysis, ideally considering three points in time (to first check for stability of the profile affiliation and then consider traits/behaviors to derive causality) would be a way to test a causal relationship (Lanza et al., 2013). Conducting a Latent Transition Analysis of followers’ role orientations and their behaviors may therefore be an avenue for future research.

Additionally, regarding the sample structure, a potential non-response bias may have led to followers of the Moderate Anti-Prototype and the Moderate Prototype profiles being (at least slightly) overrepresented. An indication of this mechanism can be seen in the dropout from *t*_1_ to *t*_2_. The dropout suggests that followers with low personal initiative may be more likely not to respond (however, this indication needs to be taken with caution, considering the overall small size of the Anti-Prototype profile). A similar phenomenon has been found regarding conscientiousness, although findings were mixed (Rogelberg et al., 2003), and conscientiousness has most probably not been the decisive factor in this study, as otherwise more followers of the Moderate Anti-Prototype profile would not have responded either. Overall, one way to mitigate the potential non-response bias could be to prepay participants before they participate in the survey (Rose et al., 2007). Although this approach is not free of limitations either, it may then be fruitful to compare the results of a prepaid sample with the results of the present, incentivized convenience sample.

Moreover, only the three factors Enthusiasm, Industry, and Good Citizen could be considered for the LPA. Thus, for instance, no insights could be obtained on the likely relevant factor Conformity (Carsten et al., 2010). By giving recommendations for the quantitative measurement of followers’ role orientations, this paper aims to support upcoming research on additional factors of followers’ role orientations. In this way, a more refined understanding of followers’ role orientations can be generated in the future.

## Data availability statement

The datasets presented in this article are not readily available because data will be available on reasonable request. Requests to access the datasets should be directed to EG, elena.gesang@hhu.de.

## Ethics statement

Ethical review and approval was not required for the study on human participants in accordance with the local legislation and institutional requirements. The patients/participants provided their written informed consent to participate in this study.

## Author contributions

The author confirms being the sole contributor of this work, and has approved it for publication.
