# Supplementary material for: How do you see your role as a follower? A quantitative exploration of followers’ role orientation
Source: Front Psychol. 2022 Nov 18;13:952925. doi: 10.3389/fpsyg.2022.952925 (PMC9716213; doi:10.3389/fpsyg.2022.952925)
Supplement: Supplementary file 1 [file Data_Sheet_1.docx]

**Supplementary Table 1.** Pearson Correlations Followership (Anti-)prototype items t_1_

|  | Item | M | SD | 1 | 2 | 3 | 4 | 5 | 6 | 7 | 8 | 9 | 10 | 11 | 12 | 13 | 14 | 15 |
| --- | --- | --- | --- | --- | --- | --- | --- | --- | --- | --- | --- | --- | --- | --- | --- | --- | --- | --- |
| good citizen | 1 reliable | 8.81 | 1.18 | 1.00 |  |  |  |  |  |  |  |  |  |  |  |  |  |  |
|  | 2 loyal | 8.07 | 1.55 | .27** | 1.00 |  |  |  |  |  |  |  |  |  |  |  |  |  |
|  | 3 team player | 8.02 | 0.57 | .28** | .31** | 1.00 |  |  |  |  |  |  |  |  |  |  |  |  |
| enthusiasm | 4 excited | 7.10 | 1.80 | .23** | .47** | .27** | 1.00 |  |  |  |  |  |  |  |  |  |  |  |
|  | 5 happy | 7.36 | 1.74 | .31** | .41** | .36** | .64** | 1.00 |  |  |  |  |  |  |  |  |  |  |
|  | 6 outgoing | 6.40 | 2.15 | .17* | .33** | .24** | .59** | .54** | 1.00 |  |  |  |  |  |  |  |  |  |
| industry | 7 goes above   and beyond | 7.48 | 1.77 | .39** | .30** | .12 | .50** | .30** | .41** | 1.00 |  |  |  |  |  |  |  |  |
|  | 8 hardworking | 7.65 | 1.58 | .38** | .36** | .23** | .54** | .40** | .44** | .67** | 1.00 |  |  |  |  |  |  |  |
|  | 9 productive | 8.06 | 1.33 | .43** | .42** | .32** | .49** | .43** | .45** | .53** | .52** | 1.00 |  |  |  |  |  |  |
| incompetence | 10 slow | 3.17 | 1.88 | -.26** | -.08 | -.04 | -.23** | -.22** | -.23** | -.29** | -.25** | -.50** | 1.00 |  |  |  |  |  |
|  | 11 inexperi-  enced | 4.15 | 2.36 | -.17* | -.09 | .05 | -.21** | -.15* | -.19** | -.34** | -.21** | -.37** | .28** | 1.00 |  |  |  |  |
| insubor-dination | 12 bad-  tempered | 2.91 | 1.79 | -.23** | -.40** | -.21** | -.49** | -.57** | -.35** | -.22** | -.28** | -.36** | .31** | .09 | 1.00 |  |  |  |
|  | 13 arrogant | 2.67 | 1.75 | -.12 | -.13 | -.16* | .02 | -.10 | .02 | .05 | -.02 | .01 | -.05 | .02 | .16* | 1.00 |  |  |
| confor-mity | 14 easily in-  fluenced | 4.96 | 2.09 | -.03 | .06 | .11 | .07 | .03 | -.04 | .03 | .13 | -.18** | .23** | .18** | .06 | -.04 | 1.00 |  |
|  | 15 follows   trends | 5.61 | 2.12 | .05 | .16* | .15* | .23** | .18** | .23** | .16* | .26** | .16* | .05 | -.10 | .03 | .03 | .22** | 1.00 |

*n* = 211; *p < 0.05; **p < 0.01

**Supplementary Table 2.** Pearson Correlations Followership (Anti-)prototype items t_2_

|  | Item | M | SD | 1 | 2 | 3 | 4 | 5 | 6 | 7 | 8 | 9 | 10 | 11 | 12 | 13 | 14 | 15 |
| --- | --- | --- | --- | --- | --- | --- | --- | --- | --- | --- | --- | --- | --- | --- | --- | --- | --- | --- |
| good citizen | 1 reliable | 8.71 | 1.25 | 1.00 |  |  |  |  |  |  |  |  |  |  |  |  |  |  |
|  | 2 loyal | 7.71 | 1.89 | .29* | 1.00 |  |  |  |  |  |  |  |  |  |  |  |  |  |
|  | 3 team player | 7.96 | 1.56 | .27* | .35** | 1.00 |  |  |  |  |  |  |  |  |  |  |  |  |
| enthusiasm | 4 excited | 6.80 | 1.82 | .20 | .26* | .26* | 1.00 |  |  |  |  |  |  |  |  |  |  |  |
|  | 5 happy | 7.06 | 1.47 | .38** | .33** | .42** | .58** | 1.00 |  |  |  |  |  |  |  |  |  |  |
|  | 6 outgoing | 6.07 | 2.13 | .15 | .08 | .14 | .71** | .46** | 1.00 |  |  |  |  |  |  |  |  |  |
| industry | 7 goes above   and beyond | 7.64 | 1.93 | .24* | .31** | .06 | .58** | .29* | .34** | 1.00 |  |  |  |  |  |  |  |  |
|  | 8 hardworking | 7.68 | 1.58 | .49** | .25* | .04 | .43** | .36** | .30* | .62** | 1.00 |  |  |  |  |  |  |  |
|  | 9 productive | 7.75 | 1.29 | .39** | .13 | .27* | .39** | .38** | .21 | .50** | .55** | 1.00 |  |  |  |  |  |  |
| incompetence | 10 slow | 3.23 | 1.71 | -.34** | -.15 | -.06 | -.22 | -.17 | -.26* | -.37** | -.33** | -.48** | 1.00 |  |  |  |  |  |
|  | 11 inexperi-  enced | 4.43 | 2.15 | -.06 | -.03 | -.15 | -.05 | -.13 | -.07 | -.27* | -.12 | -.26* | .38** | 1.00 |  |  |  |  |
| insubor-dination | 12 bad-  tempered | 2.96 | 1.74 | -.30* | -.57** | -.32* | -.51** | -.49** | -.36** | -.43** | -.48** | -.41** | .45** | .21 | 1.00 |  |  |  |
|  | 13 arrogant | 2.74 | 1.69 | -.25* | -.11 | -.53** | -.09 | -.24 | -.05 | .17 | -.01 | -.10 | .07 | .06 | .23 | 1.00 |  |  |
| confor-mity | 14 easily in-  fluenced | 4.99 | 2.06 | -.05 | -.20 | -.14 | .31** | -.20 | -.27* | -.13 | -.16 | -.27* | .34** | .32** | .34** | .12 | 1.00 |  |
|  | 15 follows   trends | 5.33 | 2.06 | .16 | .16 | .11 | -.02 | .16 | .02 | .01 | .10 | -.02 | .15 | .24* | .07 | -.01 | .33** | 1.00 |

*n* = 69; *p < 0.05; **p < 0.01

**Supplementary Table 3.** Scales used and their respective items

| scale | author(s)/source | items (in German) | items (in English) |
| --- | --- | --- | --- |
| agreeableness | Rammstedt and John, 2005 | Ich neige dazu, andere zu kritisieren. (r) | I tend to criticize others. (r) |
|  | (p. 197, German) | Ich schenke anderen leicht Vertrauen, glaube an das Gute im Menschen. | I trust others easily and believe in the good in people. |
|  |  | Ich kann mich kalt und distanziert verhalten. (r) | I can be cold and aloof. (r) |
|  |  | Ich kann mich schroff und abweisend anderen gegenüber verhalten. (r) | I can be brusque and dismissive of others. (r) |
| conscientiousness | Rammstedt and John, 2005 | Ich erledige Aufgaben gründlich. | I complete tasks thoroughly. |
|  | (pp. 197-198, German) | Ich bin bequem, neige zur Faulheit. (r) | I am (lazy,) prone to laziness. (r) |
|  |  | Ich bin tüchtig und arbeite flott. | I am efficient and work fast. |
|  |  | Ich mache Pläne und führe sie auch durch. | I make plans and carry them out. |
| core-self-evaluation traits | Stumpp et al., 2010 (p. 700, German and English) | Ich bin zuversichtlich, im Leben den Erfolg zu bekommen, den ich verdiene. | I am confident I get the success I deserve in life. |
|  |  | Manchmal bin ich deprimiert. (r) | Sometimes I feel depressed. (r) |
|  |  | Wenn ich mich anstrenge, bin ich im Allgemeinen erfolgreich. | When I try, I generally succeed. |
|  |  | Wenn ich etwas nicht schaffe, fühle ich mich manchmal wertlos. (r) | Sometimes when I fail I feel worthless. (r) |
|  |  | Ich erledige Aufgaben erfolgreich. | I complete tasks successfully. |
|  |  | Manchmal habe ich das Gefühl, keine Kontrolle über meine Arbeit zu haben. (r) | Sometimes, I do not feel in control of my work. (r) |
|  |  | Im Großen und Ganzen bin ich mit mir zufrieden. | Overall, I am satisfied with myself. |
|  |  | Ich zweifle an meinen Fähigkeiten. (r) | I am filled with doubts about my competence. (r) |
|  |  | Ich bestimme, was in meinem Leben geschehen soll. | I determine what will happen in my life. |
|  |  | Ich habe das Gefühl, den Erfolg meiner Karriere nicht unter Kontrolle zu haben. (r) | I do not feel in control of my success in my career. (r) |
|  |  | Ich bin in der Lage, die meisten meiner Probleme zu bewältigen. | I am capable of coping with most problems. |
|  |  | Es gibt Zeiten, in denen mir die Dinge ziemlich düster und hoffnungslos erscheinen. (r) | There are times when things look pretty bleak and hopeless to me. (r) |
| extraversion | Rammstedt and John, 2005 | Ich bin eher zurückhaltend, reserviert. (r) | I am rather reserved. (r) |
|  | (p. 197, German) | Ich bin begeisterungsfähig und kann andere leicht mitreißen. | I am enthusiastic and can easily carry others along. |
|  |  | Ich bin eher der „stille Typ“, wortkarg. (r) | I am rather the "quiet type", taciturn. (r) |
|  |  | Ich gehe aus mir heraus, bin gesellig. | I am outgoing and sociable. |
| helpfulness towards | Staufenbiel and Hartz, 2000 (p. 83, German) | Ich ergreife freiwillig die Initiative, neuen Kollegen/Kolleginnen bei der Einarbeitung zu helfen. | I voluntarily take the initiative to help new colleagues get acquainted with their work. |
| colleagues |  | Ich helfe anderen, wenn diese mit Arbeit überlastet sind. | I help others when they are overloaded with work. |
|  |  | Ich bemühe mich aktiv darum, Schwierigkeiten mit Kollegen/Kolleginnen vorzubeugen. | I actively try to prevent trouble with colleagues. |
|  |  | Ich wirke bei auftretenden Meinungsverschiedenheiten ausgleichend auf Kollegen/Kolleginnen ein. | I have a peasing effect on colleagues when disagreements arise. |
|  |  | Ich ermuntere Kollegen/Kolleginnen, wenn diese niedergeschlagen sind. | I encourage colleagues when they are depressed. |
| implicit | Sy, 2010 (p. 76, English) | geht über das Geforderte hinaus | goes above and beyond |
| followership |  | hart arbeitend | Hardworking |
| theories |  | produktiv | Productive |
|  |  | begeistert | excited |
|  |  | fröhlich | happy |
|  |  | aus sich herausgehend | outgoing |
|  |  | zuverlässig | reliable |
|  |  | loyal | loyal |
|  |  | Teamspieler | team player |
|  |  | leicht zu beeinflussen | easily influenced |
|  |  | folgt Trends | follows trends |
|  |  | ungebildet | uneducated |
|  |  | langsam | slow |
|  |  | unerfahren | inexperienced |
|  |  | unhöflich | rude |
|  |  | schlecht gelaunt | bad temper |
|  |  | arrogant | arrogant |
|  |  | (/) | (soft spoken) |
| personal initiative | Frese et al., 1997 (p. 161, | Ich gehe Probleme aktiv an. | I actively attack problems. |
|  | English) | Immer, wenn etwas schief geht, suche ich unmittelbar nach einer Lösung. | Whenever something goes wrong, I search for a solution immediately. |
|  |  | Wenn es die Möglichkeit gibt, sich aktiv zu engagieren, nutze ich sie. | Whenever there is a chance to get actively involved, I take it. |
|  |  | Ich ergreife die Initiative, auch wenn andere es nicht machen. | I take initiative immediately even when others don’t. |
|  |  | Ich nutze meine Möglichkeiten schnell, um meine Ziele zu erreichen. | I use opportunities quickly in order to attain my goals. |
|  |  | Normalerweise mache ich mehr, als ich gebeten wurde zu tun. | Usually I do more than I am asked to do. |
|  |  | Ich bin besonders gut im Realisieren von Ideen. | I am particularly good at realizing ideas. |
| social desirability | Satow, 2012 (p. 18, German) | Ich habe schon mal über andere gelästert oder schlecht über sie gedacht. (r) | I have gossiped about others or thought badly of them before |
|  |  | Ich würde niemals schlecht über einen Kollegen oder meinen Arbeitgeber reden. | I would never speak ill of a colleague or my employer. |
| voice behavior | Liu et al., 2010 (p. 201, English) | Ich entwickele und gebe Empfehlungen an meinen Vorgesetzten zu Themen, die die Organisation betreffen. | This person develops and makes recommendations to the supervisor concerning issues that affect our organization. |
|  |  | Ich äußere mich und beeinflusse meinen Vorgesetzten bei Fragen, die die Organisation betreffen. | This person speaks up and influences the supervisor regarding issues that affect the organization. |
|  |  | Ich teile meinem Vorgesetzten meine Meinungen zu Arbeitsfragen mit, auch wenn seine Meinung anders ist und er nicht mit meiner übereinstimmt. | This person communicates his or her opinions about work issues to the supervisor even if his or her opinion is different, and the supervisor disagrees with him or her. |
|  |  | Ich spreche mit meinem Vorgesetzten über neue Ideen für Projekte oder Änderungen in den Abläufen. | This person speaks to the supervisor with new ideas for projects or changes in procedures. |
|  |  | Ich mache meinem Vorgesetzten konstruktive Vorschläge zur Verbesserung seiner Arbeit. | This person gives constructive suggestions to the supervisor to improve the supervisor's work. |
|  |  | Ich weise meinen Vorgesetzten darauf hin, redundante oder unnötige Verfahren abzuschaffen. | This person points out to his or her supervisor to eliminate redundant or unnecessary procedures. |
|  |  | Wenn mein Vorgesetzter Fehler in seiner Arbeit gemacht hat, würde ich ihn darauf hinweisen und ihm helfen, diese zu korrigieren. | If his or her supervisor made mistakes in his or her work, this person would point them out and help the supervisor correct them. |
|  |  | Ich versuche, meinen Vorgesetzten davon zu überzeugen, organisatorische Regeln oder Richtlinien zu ändern, die unproduktiv oder kontraproduktiv sind. | This person tries to persuade his or her supervisor to change organizational rules or policies that are nonproductive or counterproductive. |
|  |  | Ich schlage meinem Vorgesetzten vor, neue Strukturen, Technologien oder Ansätze zur Effizienzsteigerung einzuführen. | This person suggests his or her supervisor to introduce new structures, technologies, or approaches to improve efficiency. |
